# Critical roles of sphingosine kinase 1 in the regulation of neuroinflammation and neuronal injury after spinal cord injury

**DOI:** 10.1186/s12974-021-02092-4

**Published:** 2021-02-18

**Authors:** Chenjian Wang, Tianzhen Xu, Brittany Bolduc Lachance, Xiqiang Zhong, Guangjie Shen, Tao Xu, Chengxuan Tang, Xiaofeng Jia

**Affiliations:** 1 Department of Orthopaedics, The Third Affiliated Hospital of Wenzhou Medical University, Rui’an People’s Hospital, Wenzhou, 325200 Zhejiang China; 2 Department of Orthopaedics, Zhu’ji People’s Hospital, Shaoxing, 311800 Zhejiang China; 3 Program in Trauma, Department of Neurology, University of Maryland School of Medicine, Baltimore, MD 21201 USA; 4 Department of Neurosurgery, University of Maryland School of Medicine, 10 South Pine Street, MSTF Building 823, Baltimore, MD 21201 USA; 5 Department of Orthopaedics, University of Maryland School of Medicine, Baltimore, MD 21201 USA; 6 Department of Anatomy and Neurobiology, University of Maryland School of Medicine, Baltimore, MD 21201 USA; 7 Department of Biomedical Engineering, The Johns Hopkins University School of Medicine, Baltimore, MD 21205 USA; 8 Department of Anesthesiology and Critical Care Medicine, The Johns Hopkins University School of Medicine, Baltimore, MD 21205 USA

**Keywords:** Sphk1, Spinal cord injury, M1 microglia, NF-κB p65 activation, S1P/S1PR3/p38 MAPK pathway

## Abstract

**Background:**

The pathological process of traumatic spinal cord injury (SCI) involves excessive activation of microglia leading to the overproduction of proinflammatory cytokines and causing neuronal injury. Sphingosine kinase 1 (Sphk1), a key enzyme responsible for phosphorylating sphingosine into sphingosine-1-phosphate (S1P), plays an important role in mediating inflammation, cell proliferation, survival, and immunity.

**Methods:**

We aim to investigate the mechanism and pathway of the Sphk1-mediated neuroinflammatory response in a rodent model of SCI. Sixty Sprague-Dawley rats were randomly assigned to sham surgery, SCI, or PF543 (a specific Sphk1 inhibitor) groups. Functional outcomes included blinded hindlimb locomotor rating and inclined plane test.

**Results:**

We discovered that Sphk1 is upregulated in injured spinal cord tissue of rats after SCI and is associated with production of S1P and subsequent NF-κB p65 activation. PF543 attenuated p65 activation, reduced inflammatory response, and relieved neuronal damage, leading to improved functional recovery. Western blot analysis confirmed that expression of S1P receptor 3 (S1PR3) and phosphorylation of p38 mitogen-activated protein kinase (p38 MAPK) are activated in microglia of SCI rats and mitigated by PF543. In vitro, we demonstrated that Bay11-7085 suppressed NF-κB p65 and inhibited amplification of the inflammation cascade by S1P, reducing the release of proinflammatory TNF-α. We further confirmed that phosphorylation of p38 MAPK and activation of NF-κB p65 is inhibited by PF543 and CAY10444. p38 MAPK phosphorylation and NF-κB p65 activation were enhanced by exogenous S1P and inhibited by the specific inhibitor SB204580, ultimately indicating that the S1P/S1PR3/p38 MAPK pathway contributes to the NF-κB p65 inflammatory response.

**Conclusion:**

Our results demonstrate a critical role of Sphk1 in the post-traumatic SCI inflammatory cascade and present the Sphk1/S1P/S1PR3 axis as a potential target for therapeutic intervention to control neuroinflammation, relieve neuronal damage, and improve functional outcomes in SCI.

## Introduction

Spinal cord injury (SCI) affects approximately 282,000 people in the USA, with 17,000 new cases each year [1]. Less than 1% of patients recover full neurological function at the time of discharge, and despite advances in science, life expectancy for SCI patients is significantly lower than the general population, and there has been minimal change in mortality rate for these patients over the last 40 years [1]. Current clinical interventions such as surgical decompression, drug therapies, and hypothermia are aimed at reduction of secondary injury and remain non-curative [2], and the care costs of this disease remain immense [3]. Secondary neurological injury in the form of cell necrosis and tissue loss persist for several weeks after initial injury, which provides a window for potential intervention with neuroprotective strategies. Lack of targeted drug therapies for the mitigation of permanent injury and for improved neurological functional recovery in this disease process presents a critical gap in modern medicine. Historically, SCI drug therapies for the manipulation of the inflammatory response, such as methylprednisolone, have not been effective and may even have deleterious consequences [2]. In order to address this interventional gap, it is imperative that we further understand the complex cascade mechanisms involved in secondary tissue damage of SCI patients.

Though knowledge of the complex pathological cascades that contribute to secondary tissue damage of the CNS grows, the inflammatory response primarily mediated by macrophages in the neurological recovery process remains controversial [4, 5]. Microglia, the resident macrophages of the innate neuroimmune system, are rapidly activated after SCI, and this activation remains sustained for weeks to months [6]. Microglia manifest multiple phenotypes in response to cues from the microenvironment and depending on the nature of tissue injury [4, 7]. These different phenotypes have dramatically different effects on the modulation of CNS repair and regeneration. Microglia polarized to the M1 phenotype are proinflammatory and rapidly induce the synthesis of proinflammatory cytokines and other inflammatory mediators following acute injury, such as in SCI. Released molecules such as interleukin 1β and tumor necrosis factor (TNF) promote the degeneration and apoptosis of neurons [7–9], resulting in secondary CNS injury.

Sphingosine kinases (Sphks) are the rate-limiting catalyzing enzymes that convert sphingosine into sphingosine-1-phosphate (S1P), an important lipid signal conductor known to regulate physiological processes such as cell growth, death, senescence, adhesion, migration, inflammation, and angiogenesis [10–12]. Previous studies suggest that Sphk-derived S1P is linked to multiple disease processes via the “inside-out” signaling pathway [13]. Activation of Sphk1 leads to the intracellular production of S1P, which is necessary for IkBa degradation and NF-κB activation. These responses are mediated by intracellular S1P, independently of a family of five cell surface G-protein-coupled receptors (S1PR 1-5) [14], and these receptors are expressed widely throughout the body. S1P can also be transported out of the cell and bind S1P receptors, signaling extracellularly via autocrine or paracrine pathways [13]. A recent study reported that S1P is generated during central nervous system injury, inflicted by penetrating stab wound to the brain, and can activate surface receptors on astrocytes to promote neuroinflammation amplification and increase nuclear translocation of NF-κB [15], demonstrating that S1P can function intracellularly as a second messenger or act extracellularly by binding to surface S1P receptors.

The Sphk1/S1P/S1PR axis has been a proposed therapeutic target for cancer and inflammatory diseases [10]. However, the mechanisms and pathways of Sphk1/S1P/S1PRs participating in neuroinflammation within the CNS have not been thoroughly elucidated, and this pathway has not yet been investigated or implicated in traumatic SCI. In addition, the diverse roles of this axis in multiple signaling pathways throughout the body has complicated therapeutics and places an emphasis on the need for highly specific targeting to avoid side effects [10]. In progressive heart failure, S1P stimulate phosphorylation of p38 MAPK signaling, leading to increased myogenic response [16]. A recent study showed that S1PR3 expression is upregulated in pericytes after SCI, and S1P/S1PR3 mediates the proliferation of pericytes via the Ras/pERK pathway [17]. Neural cells, including microglia, were found to express S1P receptors (S1PRs), which may be a target of S1P in the CNS [18]. Microglia polarization plays a major role in the inflammatory response of the nervous system, and S1P3 has been identified as a mediator in the modulation of microglial activation and M1 polarization [19]. Although much of the research has focused on the Sphk1/S1P/S1PR axis, the roles of this axis in multiple signaling pathways are very complicated, and the role of microglia within this pathway, particularly after SCI, has yet to be explored. We chose to focus our study on the downstream effects of microglial S1PR3 activation, given the demonstration of this receptor on microglia and the prior implication of this receptor inpost-ischemic inflammation [19]. We hypothesize that this pathway plays a key role in secondary neuroinflammation after acute traumatic spinal cord injury and that targeting this pathway can mediate inflammation, neuronal apoptosis, and improve neurologic recovery.

This study identifies the role of the Sphk1/S1P mechanism and pathway in SCI. We found that microglia were activated and predominantly polarized to the M1 phenotype in the injured spinal cord tissue of rats in vivo and under LPS stimulation in vitro. In addition, we demonstrated that Sphk1 expression is upregulated in activated microglia and that inhibition of Sphk1 attenuates the inflammatory response and relieves neuronal damage facilitated by M1 microglia, ultimately reducing the downstream activation of p38 MAPK and NF-κB. Functional outcomes after traumatic SCI improved with targeted inhibition of the Sphk1/S1P pathway. This study demonstrates that the Sphk1/S1P/S1PR3 axis participates in a specific pathway that regulates neuroinflammation in traumatic SCI and opens the door to a therapeutic target for mitigating neuronal injury and improving neurological outcome in a disease process for which there are currently no targeted pharmacologic interventions.

## Methods

### Reagents and antibodies

Cell counting kit-8 (CCK-8) was obtained from Dojindo (Kumamoto, Japan). TNF-α ELISA Kit was purchased from R&D Systems (Minneapolis, MN, USA). BAY11-7085 and SB203580 were purchased from Selleck Chemicals (Houston, TX, USA). LPS, PF543 hydrochloride, and S1P (C18H38NO5P, CAS#:26993-30-6) were purchased from Sigma-Aldrich (St Louis, MO, USA). CAY10444 was purchased from Cayman Chemical (Ann Arbor, MI). Recombinant Human β-NGF was purchased from Peprotech (USA). Monoclonal antibodies specific for cleaved caspase-3, COX-2, phospho-p38 MAPK, phospho-NF-κB p65, NF-κB p65, and IκB were procured from Cell Signaling Technologies (Beverly, MA, USA). Polyclonal antibodies specific for Iba-1, NeuN, iNOS, IL-6, TNF-α, Bax, and p38 MAPK and a monoclonal antibody specific for GAPDH were purchased from Proteintech (Wuhan, China). Polyclonal antibodies specific for Bcl-2 and Sphk1 and a monoclonal antibody specific for S1PR3 were purchased from Abcam (Cambridge, UK). AlexaFluor 568, AlexaFluor 488 donkey anti-rabbit/mouse, and horseradish peroxidase-labeled secondary antibodies were purchased from Abcam. 4′, 6-Diamidino-2-phenylindole (DAPI) was obtained from Yeasen Biotech (Shanghai, China). For the in vitro studies, PF543 hydrochloride was dissolved in dimethyl sulfoxide (DMSO) and was diluted appropriately with cell culture medium (final DMSO concentration ≤ 1‰). Bay 11-7085 (inhibitor of NF-κB) was dissolved in DMSO and was diluted into the concentration of 5 μM. SB203580 (inhibitor of P38 MAPK) was dissolved in DMSO and was diluted into the concentration of 10 μM. CAY10444 (inhibitor of S1P3) was dissolved in DMSO [19] and was diluted into appropriate concentration of 10 μM [20]. S1P was dissolved in methanol according to the manufacturer’s instructions and was diluted into the concentration of 1 μM [20]. LPS was dissolved in PBS and was diluted with cell culture medium into the concentration of 1 μg/ml [21]. For the in vivo studies, PF543 hydrochloride was dissolved in normal saline. Per the manufacturer’s instructions, PF543 hydrochloride is soluble in both aqueous solutions and DMSO. Since our in vivo experiments used higher doses and DMSO is toxic to animals, we chose to use saline in these experiments. Prior studies have similarly substituted PBS as a solvent to reduce toxic side effects of the PF543 vehicle [22, 23]

### Spinal cord injury model and drug treatment

A total of 60 adult female Sprague-Dawley rats (200–250 g) were acquired from the Charles River Laboratory Animal Company in Shanghai, China, and were randomly divided into three groups: Sham (*n* = 19), SCI (*n* = 25), and PF543 group (*n* = 16). All rats were housed under controlled environmental conditions. The care and use protocols of all animals were conducted according to the guidelines set forth by the Chinese National Institutes of Health. To induce SCI, the rats were anesthetized with 4% chloral hydrate (3 ml/kg) via intraperitoneal injection. Then, we performed a laminectomy at the T9 vertebra. After fully exposing the spinal cord, a vascular clip (30 g forces, Oscar, Shanghai, China) was used to induce moderate crush injury for 1 min, as we previously published [24]. For sham-operated rats, the same surgical procedure was performed except for compression. Postoperatively, the animals’ urinary bladders were emptied twice daily. After surgery, PF-543 was immediately administered by intraperitoneal injection [22, 23] and was repeated daily at a dose of 10 mg/kg until the rats were sacrificed. As controls, animals in the sham and SCI groups were injected intraperitoneally with an equivalent volume of normal saline.

### Behavioral analysis

The Basso, Beattie, and Bresnahan (BBB) locomotion rating scale [25] and the inclined plane test were conducted 1, 3, 7, 14, and 21 days following surgery. The BBB score evaluated hindlimb locomotor function on a scale from 0 to 21, with 0 representing no observable movement and 21 representing normal movement, as we previously published [24, 26]. The inclined plane test was investigated using a board secured in place at one end with the free edge of the board gradually raised to increase the angle of the incline. The maximum angle at which the rats maintained stability for at least 5 s was recorded as the inclined plane test angle [24]. The outcome measures were assessed by three independent examiners blinded to the experimental conditions.

### Histology and immunofluorescence

Rats were sacrificed with 8% chloral hydrate (3.5 ml/kg, i.p.) at specific time-points after SCI and then perfused with normal saline. For the H&E staining, Nissl staining and immunofluorescence analysis, tissue segments containing the spinal cord lesion (1 cm on each side of the lesion) and spinal cord sections (0.5-cm) from each specimen were dissected out, post-fixed with 4% paraformaldehyde for 6 h, and then embedded in paraffin. Transverse sections (5 μm thick) were mounted on slides following staining. Histopathological examinations were performed by H&E and Nissl staining, according to the manufacturer’s instructions. Bright-field images were acquired using light microscopy. The transverse sections were treated with the following primary antibodies: anti-Sphk1 (1:100), anti-S1PR3 (1:100), anti-Iba-1 (1:200), anti-NeuN (1:100), and anti-cleaved caspase 3 (1:400) before being washed four times with PBS and incubated with AlexaFluor 568 and AlexaFluor 488 donkey anti-rabbit/mouse secondary antibodies for 1 h at 37 °C. Sections were then washed with PBS, incubated with DAPI for 1 min, rinsed with PBS, and finally sealed with a coverslip. All images were captured on a fluorescence microscope.

### Cell culture

The murine HAPI microglial cell line was purchased from Otwo Biotech (Shenzhen, China). The PC12 cell line was purchased from the Shanghai Institute of Cell Biology and has been differentiated in vitro by 5% horse serum and NGF (50 ng/ml) to acquire neuronal features, which were confirmed via a microscope. HAPI cells and PC12 cells cultured in DMEM medium (Gibco BRL Co., Ltd., USA) supplemented with 10% fetal bovine serum (Gibco BRL Co., Ltd., USA), 100 units/ml penicillin, and 100 μg/ml streptomycin, were incubated at 37 °C in a humidified atmosphere containing 5% CO_2_ and were passaged every 3 days.

### HAPI cell treatment

To establish the HAPI inflammation model, we added LPS (1 μg/ml) to the HAPI culture medium for 24 h. To study the mechanism of the Sphk1-mediated inflammatory response in HAPI cells, we pretreated the cells with different doses of PF543 for 12 h before treating them with LPS (1 μg/ml) alone or LPS with S1P (1 μM). We also pretreated the cells with 5 μM BAY 11-7085 (an inhibitor of NF-κB activation), 10 μM CAY10444 (a selective antagonist of S1P binding to S1PR3), and 10 μM SB203580 (an inhibitor of p38 MAPK activation) for 2 h before treating them with different stimuli. All experiments were performed in triplicate.

### Microglia/neuron coculture

To mimic the environment and investigate the relationship between activated microglia and neuronal survival, we utilized a transwell coculture system (Corning, 0.4-μm pores, USA) [27]. HAPI and PC12 cells, which have been used as substitutes for primary cells in vitro as they retain many of the morphological and functional characteristics of primary microglia and neurons, were separately seeded into 12-well (3 × 10^5^ HAPI cells/insert and 5 × 10^5^ PC12 cells/well) transwell plates for the coculture experiment. The HAPI cells were pretreated with or without 100 nM PF543 for 12 h before stimulation with 1 μg/ml LPS for another 24 h. After stimulation, the HAPI cell inserts were rinsed with PBS to eliminate the effects of any remaining LPS and PF543 before being placed into the PC12 wells. After 24 h of coculture, we measured neuronal apoptosis by TUNEL assay and Western blot.

### Cell viability measurements

Cell viability was assessed with a cell counting kit-8 assay according to the manufacturer’s protocol. Briefly, HAPI cells were seeded in 96-well plates (5000 cells/well) and cultured in DMEM with 10% FBS at 37 °C and 5% CO_2_ for 24 h. Then, different doses of PF543 (0, 5, 10, 50, 100, and 500 nM and 1, 5, and 10 μM) were added. After treatment, the cells were washed with phosphate-buffered saline (PBS), 100 μl of DMEM containing 10 μl of CCK-8 solution was added to each well, and the plate was incubated for an additional 2 h. The absorbance of the wells was measured at 450 nm using a microplate reader (Thermo Scientific, Multiskan Go, Waltham, MA, USA).

### ELISA for measurement of TNF-α

The inflammatory cytokines tumor necrosis factor-α (TNF-α) in the cell culture supernatants were measured using commercial ELISA Kits following the manufacturer’s instructions.

### Cellular immunofluorescence

For immunofluorescence analysis, the samples were fixed with 4% paraformaldehyde for 15 min, permeabilized with 0.5% (v/v) Triton X-100 for 10 min, and then blocked with 1% (w/v) goat serum albumin for 1 h at 37 °C. The slides were incubated at 4 °C overnight with primary antibodies against Sphk1 (1:100), COX-2 (1:400), TNF-α (1:400), NF-κB p65 (1:200), and phospho-p38 (1:1600). The following day, the slices were washed three times with PBS and then incubated with AlexaFluor 568 and AlexaFluor 488 donkey anti-rabbit/mouse secondary antibodies for 1 h at 37 °C before being labeled with DAPI for 1 min at room temperature. Finally, three fields of view per slide were randomly selected for observation under a fluorescence microscope, and staining intensities were measured by observers who were blinded to the experimental groups using ImageJ software (National Institutes of Health, Bethesda, MD) [28].

### TUNEL analysis

The TUNEL method is useful for measuring apoptotic DNA fragmentation. PC12 cells were seeded on slides in a 24-well plate and allowed to attach for 24 h. Then, HAPI cells were pretreated with or without 0.1 μM PF543 for 12 h before being treated with LPS and placed in the wells containing the PC12 cells for 24 h. Next, the PC12 cells were washed gently with PBS twice and fixed with freshly prepared 4% paraformaldehyde for 30 min before being incubated with 0.1% Triton X-100 for 5 min. The cells were washed with PBS three times after each step. The cells were then stained using the in situ cell death detection kit, fluorescein (Roche, USA), and DAPI, according to the manufacturer’s instructions. Apoptosis was measured using a fluorescence microscope (Olympus, Tokyo, Japan).

### Western blot analysis

For in vivo protein analysis, a spinal cord segment at the lesion epicenter was dissected at 3 days and immediately stored at − 80 °C. Proteins isolated from animal tissue or PC12 cells were first quantified with BCA reagents. Then, cellular samples containing 30 μg of protein and tissue samples containing 60 μg of protein were separated by SDS-PAGE before being transferred to PVDF membranes. Western blot was performed using standard protocols as we previously described [29]. Membranes were blocked with 5% (w/v) milk (Bio-Rad) in TBS with 0.05% Tween 20 (TBST) for 2 h at room temperature and then incubated with the appropriate primary antibodies overnight. Next, the membranes were washed with TBST 3 times and incubated with horseradish peroxidase-conjugated secondary antibodies for 2 h at room temperature (RT). All signals were detected by a ChemiDocXRS + Imaging System (Bio-Rad). Densitometric analysis of all Western blot bands was conducted, with densities normalized to those of GAPDH. The gray value was measured and analyzed by Image Lab (Bio-Rad).

### Statistical analysis

Numerical data from at least three individual experiments are presented as mean ± SEM. One-way analysis of variance (one-way ANOVA) followed by Tukey’s post hoc analysis or Kruskal–Wallis analysis (non-parametric ANOVA) plus Dunn’s multiple comparisons (when the data failed the assumptions of one-way ANOVA) were used to test differences between groups at specific times. Between-group differences in BBB scores and inclined plane test results were analyzed using repeated measurement two-way mixed ANOVA, followed by Bonferroni’s test. Statistical significance was established as *P < 0.05 and **P < 0.01 versus the indicated group.

## Results

### Inhibition of Sphk1 attenuated the neuroinflammation and neuronal apoptosis and also improved the functional recovery in SCI rats

**Fig. 1:**
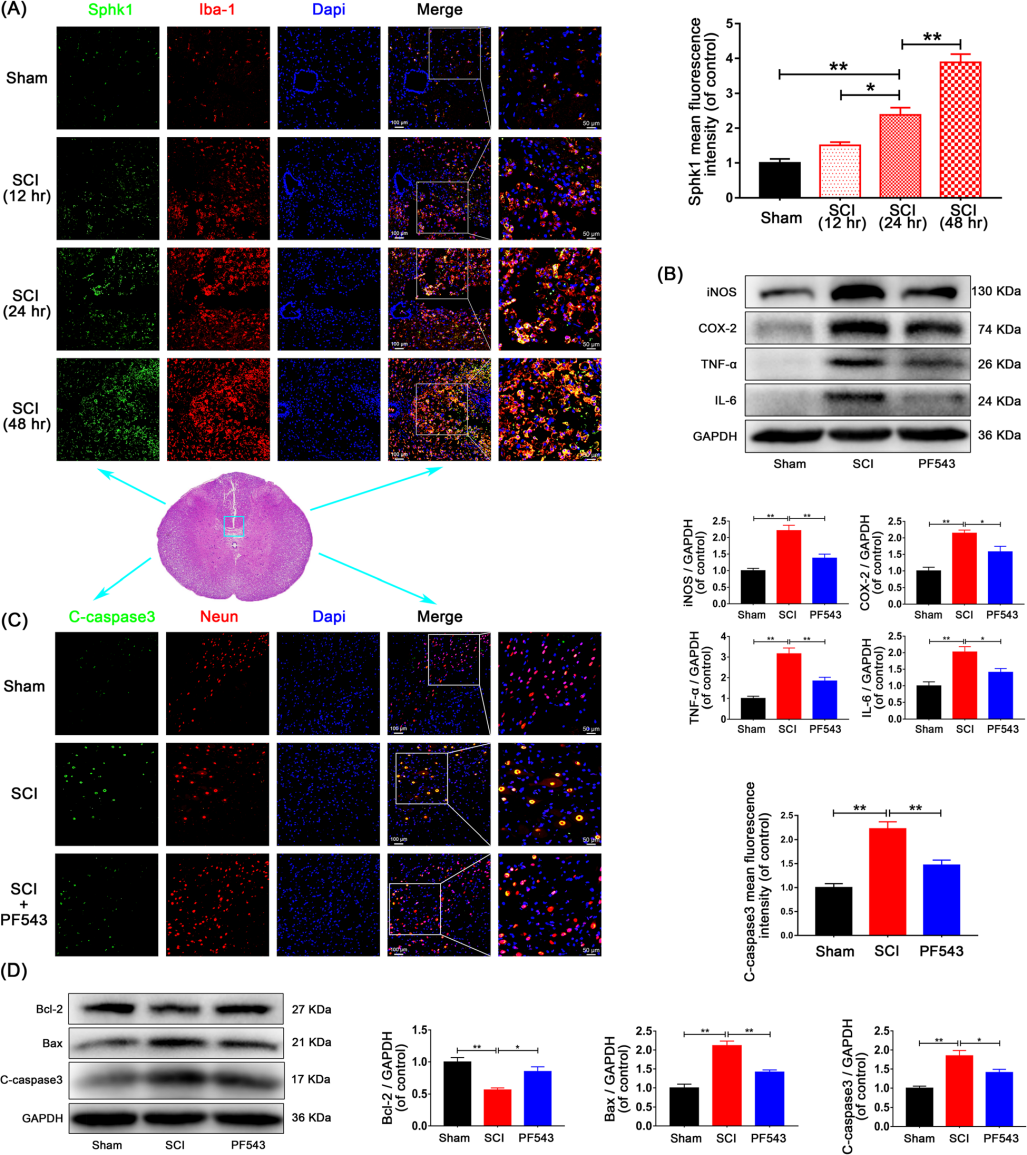
Inhibition of Sphk1 attenuates microglial M1 polarization and prevents neuronal apoptosis in SCI rats. **a** Double-staining for Sphk1 (green)/Iba-1 (red) in injured spinal cord tissue from each group of rats (scale bar: 100 μm or 50 μm; 5 mm caudal from injury core). Sphk1 was induced over time in activated microglia of SCI rats. **b** Western blot analysis of iNOS, COX-2, TNF-α, IL-6, and GAPDH expression in each group of rats. M1 polarization and inflammatory mediator release was suppressed by PF543 treatment. **c** Representative Western blots of cleaved caspase 3, Bcl-2, Bax, and GAPDH expression in each group of rats. PF543 prevented SCI-induced neuronal apoptosis. **d** Double-staining for cleaved caspase 3 (green)/NeuN (red) in sections of injured spinal cord tissue from each group of rats (scale bar: 100 μm or 50 μm). *n* = 3 per group. *P < 0.05 and **P < 0.01

To verify post-SCI inflammation and the induction of Sphk1 in a rat model of SCI, spinal cord tissue was harvested after SCI for double-immunofluorescence assays. The results showed enhanced staining of Sphk1 over time in activated microglia (Fig. 1a, Sham group: 1.00 ± 0.11 vs. SCI-24 h group: 2.38 ± 0.21, *P* = 0.002, SCI-12 h group: 1.51 ± 0.09 vs. SCI-24 h group: 2.38 ± 0.21, *P* = 0.030, SCI-24 h group: 2.38 ± 0.21 vs. SCI-48 h group: 3.89 ± 0.24, *P* = 0.001). To investigate the role of Sphk1 following SCI, we examined the effects of PF543, a highly selective and efficient inhibitor of Sphk1 leading to reduced intracellular S1P [30], on microglial M1 polarization and neuronal apoptosis 3 days after surgery. Western blot assays for additional markers showed that expression of M1 phenotypic markers iNOS and COX-2 and proinflammatory cytokines TNF-α and IL-6 were significantly increased after SCI (Fig. 1b, Sham group vs. SCI group: 1.00 ± 0.07 vs. 2.21 ± 0.16, *P* = 0.001 for iNOS, 1.00 ± 0.11 vs. 2.14 ± 0.10, *P* = 0.002 for COX-2, 1.00 ± 0.10 vs. 3.16 ± 0.28, *P* < 0.001 for TNF-α, 1.00 ± 0.12 vs. 2.02 ± 0.16, *P* = 0.004 for IL-6). Elevated expression of these markers was suppressed by PF543 administration (SCI group vs. PF543 group: 2.21 ± 0.16 vs. 1.38 ± 0.12, *P* = 0.007 for iNOS, 2.14 ± 0.10 vs. 1.58 ± 0.16, *P* = 0.043 for COX-2, 3.16 ± 0.28 vs. 1.85 ± 0.18, *P* = 0.008 for TNF-α, 2.02 ± 0.16 vs. 1.41 ± 0.11, *P* = 0.038 for IL-6).

We performed double-immunofluorescence staining for cleaved caspase 3 and NeuN (a neuron marker), and the results showed that the staining intensity of cleaved caspase 3 in neurons increased in the injured spinal cord segment and that PF543 treatment weakened this effect (Fig. 1c. Sham group: 1.00 ± 0.08 vs. SCI group: 2.22 ± 0.15, *P* < 0.001, SCI group: 2.22 ± 0.15 vs. PF543 group: 1.47 ± 0.10, *P* = 0.008). Moreover, analysis of expression of proteins by Western blot, demonstrated that the pro-apoptotic proteins Bax and cleaved caspase-3 significantly increased and pro-survival protein Bcl-2 decreased in SCI rats (Fig. 1d, Sham group vs. SCI group: 1.00 ± 0.07 vs. 0.56 ± 0.03, *P* = 0.005 for Bcl-2, 1.00 ± 0.10 vs. 2.12 ± 0.12, *P* < 0.001 for Bax, 1.00 ± 0.05 vs. 1.85 ± 0.14, *P* = 0.002 for cleaved caspase 3). Consistent with the post-SCI inflammatory changes, these findings were reversed and neural apoptosis was ameliorated in the PF543 treatment group (SCI group vs. PF543 group: 0.56 ± 0.03 vs. 0.85 ± 0.07, *P* = 0.034 for Bcl-2, 2.12 ± 0.12 vs. 1.42 ± 0.06, *P* = 0.005 for Bax, 1.85 ± 0.14 vs. 1.41 ± 0.08, *P* = 0.042 for cleaved caspase 3).

**Fig. 2:**
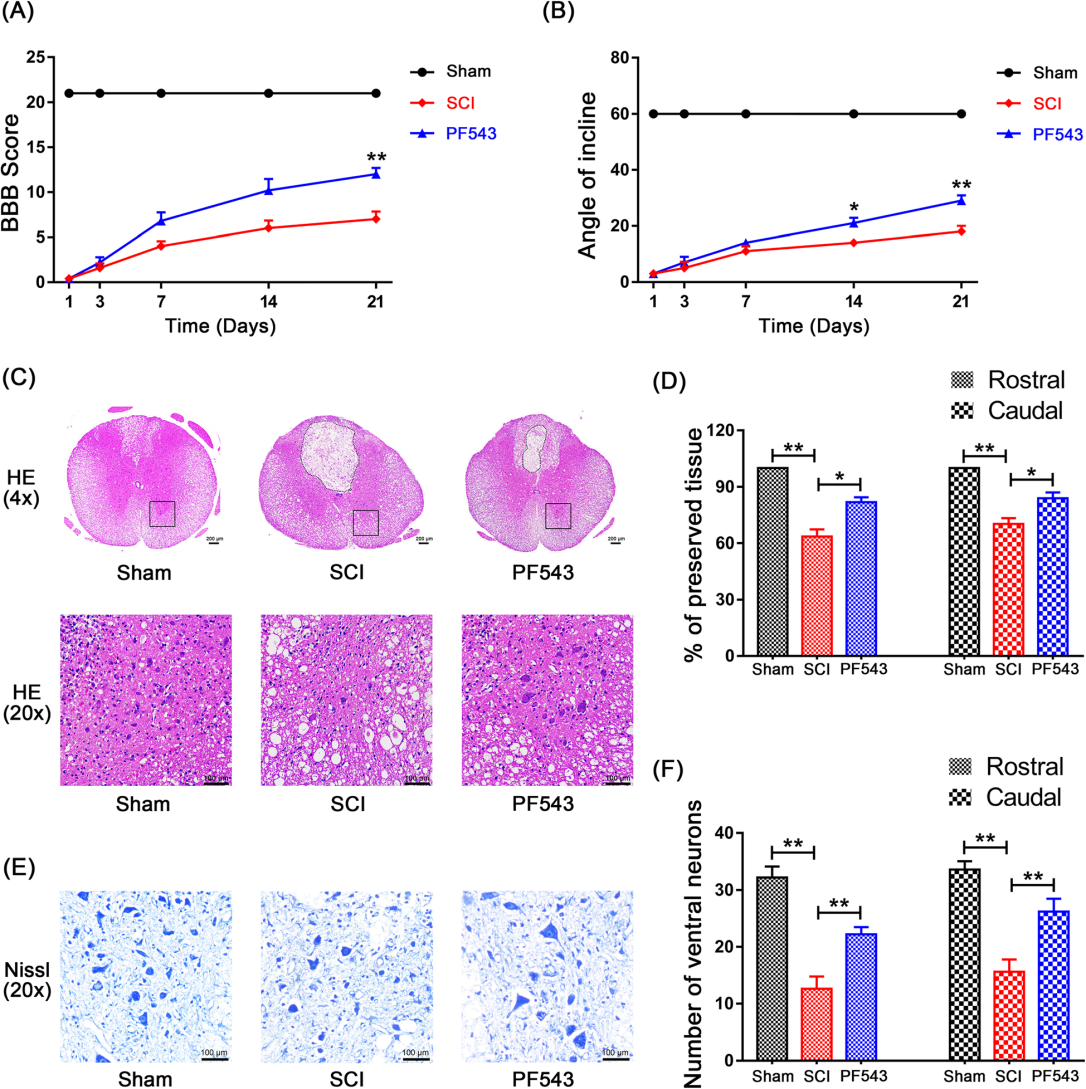
PF543 reduces structural tissue damage, reduces neuron loss, and promotes the recovery of motor function after experimental acute spinal cord injury. **a** Basso, Beattie and Bresnahan (BBB) scores. **b** Inclined plane test scores. **c** H&E stains at 7 days. Scale bars are 200 μm (× 4) and 100 μm (× 20). Dashed line area shows cavity of spinal cord. **d** Graphic presentation of the percentage of preserved tissue relative to the transverse area of the spinal cord on the seventh day after surgery. **e** Nissl staining to assess the loss of neurons at 7 days (scale bar: 100 μm). **f** Counting analysis of ventral neurons at rostral 5 mm and caudal 5 mm. *n* = 5 per group. *P < 0.05 and **P < 0.01

To evaluate the extent of post-SCI locomotion recovery, we observed behavioral changes after injury using blinded BBB scores and the inclined plane test. Rats showed no observable movement of their hindlimbs immediately following SCI, indicating that a severe SCI model was established. Throughout the course of post-SCI recovery, BBB scores and angle of incline estimates showed that the PF543 treatment group experienced greater functional recovery at 14 days post-SCI (Fig. 2a, b, 14.00 ± 1.00 vs. 21.00 ± 1.87, *P* = 0.014 for angle assessment of incline on day 14; 7.00 ± 0.84 vs. 12.00 ± 0.71, *P* = 0.004 for BBB score on day 21; 18.00 ± 2.00 vs. 29.00 ± 1.87, *P* = 0.001 for angle assessment of incline on day 21), indicating that inhibition of Sphk1 improves motor functional recovery after SCI.

In addition, we evaluated the histological morphology changes of injured spinal cord tissue in each group 7 days after surgery using H&E and Nissl staining. The results showed that rats in the SCI group exhibited severely damaged central gray matter and dorsal white matter compared with the sham group. Compared with damage in the SCI group, the PF543 treatment group exhibited attenuated damage in similar locations (Fig. 2c, d, Sham group vs. SCI group: 100 vs. 63.49 ± 3.78, *P* = 0.002 for percentage of rostral preserved tissue, 100 vs. 70.19 ± 3.06, *P* = 0.002 for percentage of caudal preserved tissue; SCI group vs. PF543 group: 63.49 ± 3.78 vs. 81.79 ± 2.60, *P* = 0.014 for percentage of rostral preserved tissue, 70.19 ± 3.06 vs. 83.91 ± 3.07, *P* = 0.037 for percentage of caudal preserved tissue), indicating the protective effects of PF543 in SCI. Moreover, we counted the number of ventral neurons at high magnification. As shown in Fig. 2e, f (Sham group vs. SCI group: 32.20 ± 1.93 vs. 12.60 ± 2.18, *P* < 0.001 for number of rostral neurons, 33.60 ± 1.47 vs. 15.60 ± 2.18, *P* < 0.001 for number of caudal neurons; SCI group vs. PF543 group: 12.60 ± 2.18 vs. 22.20 ± 1.28, *P* = 0.008 for number of rostral neurons; 15.60 ± 2.18 vs. 26.20 ± 2.29, *P* = 0.008 for number of caudal neurons), SCI induced significant neuronal loss, and PF543 treatment attenuated this phenomenon, demonstrating that PF543 can reduce neuronal loss and improve pathological injury. These data indicate that inhibition of Sphk1 attenuates microglial M1 polarization and prevents neuronal apoptosis after SCI, which are, in turn, related to improved functional recovery.

### LPS induces microglial M1 polarization and increases Sphk1 expression

**Fig. 3:**
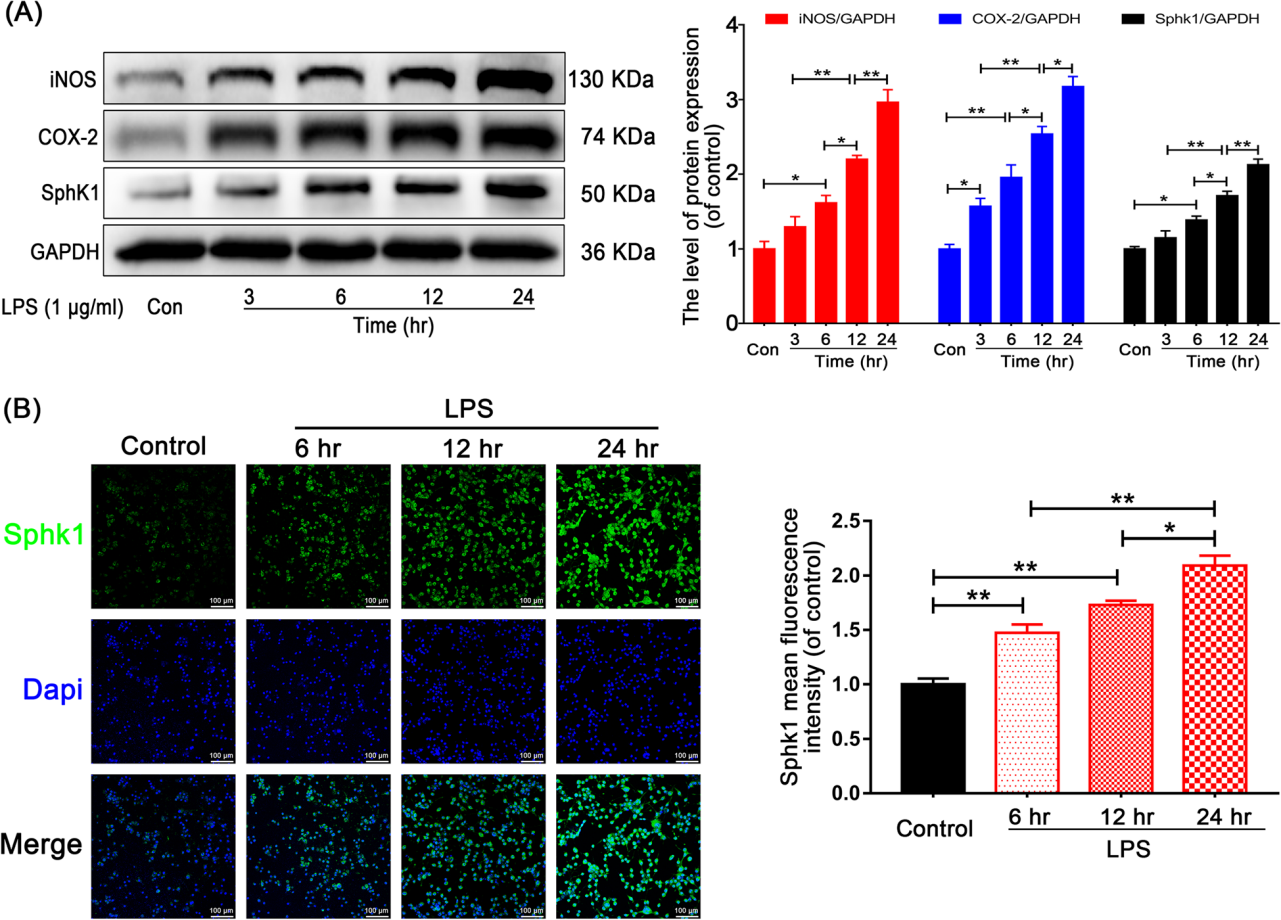
LPS induces M1 microglia polarization and Sphk1 expression in HAPI cells. **a** Representative Western blots and quantitative data for iNOS, COX-2, and Sphk1, GAPDH expression in each group of HAPI cells. LPS induced M1 microglia polarization and Sphk1 expression in a time-dependent manner. **b** Immunofluorescence staining for Sphk1 in each group of HAPI cells (scale bar: 100 μm). *n* = 3 independent experiments. *P < 0.05 and **P < 0.01

In our in vitro experiment, we performed LPS stimulation to mimic the post-SCI inflammatory response in the HAPI microglial cell line. To verify the results, we analyzed the expression of specific M1 phenotypic proteins iNOS and COX-2 by Western blot. Both markers increased in a time-dependent manner after LPS stimulation in the corresponding group (Fig. 3a, in the five groups for Control and LPS-3 h, 6 h, 12 h, and 24 h groups, respectively). The iNOS rankings were 1.00 ± 0.10 < 1.29 ± 0.14 < 1.61 ± 0.10 < 2.20 ± 0.05 < 2.97 ± 0.17, Control group vs. LPS-6 h group, *P* = 0.027; LPS-6 h group vs. LPS-12 h group, *P* = 0.035; LPS-12 h group vs. LPS-24 h group, *P* = 0.007; LPS-3 h group vs. LPS-12 h group, *P* = 0.002. The COX-2 rankings were 1.00 ± 0.06 < 1.57 ± 0.11 < 1.96 ± 0.17 < 2.54 ± 0.10 < 3.17 ± 0.13, Control group vs. LPS-3 h group, *P* = 0.043; Control group vs. LPS-6 h group, *P* = 0.001; LPS-6 h group vs. LPS-12 h group, *P* = 0.038; LPS-12 h group vs. LPS-24 h group, *P* = 0.023; LPS-3 h group vs. LPS-12 h group, *P* = 0.001). Accordingly, the expression of Sphk1 showed the same trend (Control group vs. LPS-6 h group, *P* = 0.012; LPS-6 h group vs. LPS-12 h group, *P* = 0.031; LPS-12 h group vs. LPS-24 h group, *P* = 0.007; LPS-3 h group vs. LPS-12 h group, *P* < 0.001).

Immunofluorescence of Sphk1 in LPS-stimulated microglia displayed staining intensity, which increased in a time-dependent manner (Fig. 3b, Control group: 1.00 ± 0.05 vs. LPS-6 h group: 1.47 ± 0.08, P = 0.005; LPS-12 h group: 1.73 ± 0.04 vs. LPS-24 h group: 2.09 ± 0.09, *P* = 0.023; Control group vs. LPS-12 h group, *P* < 0.001; LPS-6 h group vs. LPS-24 h group, *P* < 0.001). These findings suggest that LPS treatment can induce microglial activation and M1 polarization, which is linked to proinflammatory cytokine production and induces nerve cell apoptosis. In addition, the inflammatory amplification cascade may be partly regulated by the induction of Sphk1 in activated microglia.

### Sphk1 inhibition attenuates microglial M1 polarization

**Fig. 4:**
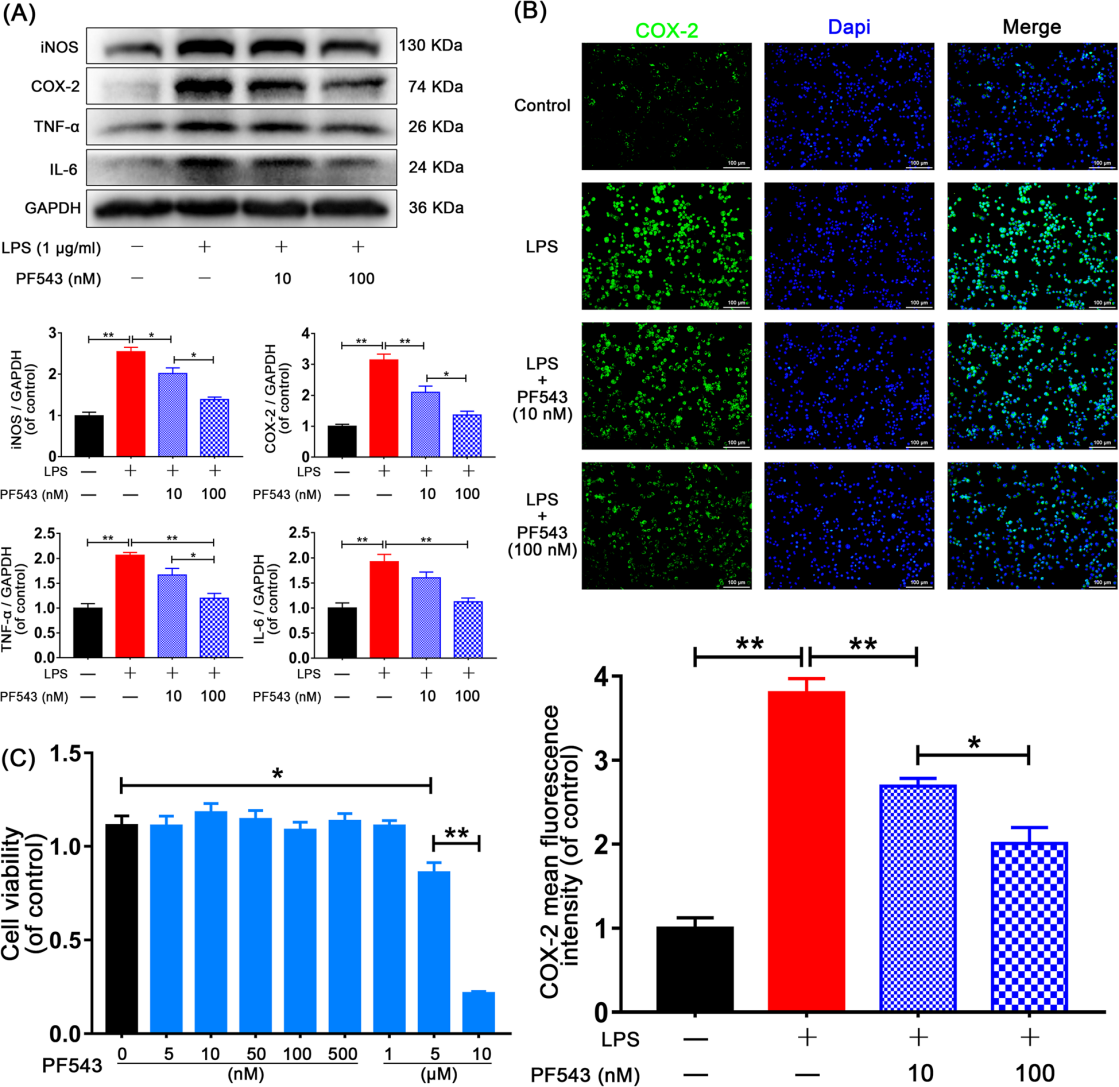
PF543 attenuates neuroinflammation in vitro. HAPI cells were pretreated with 10 nM and 100 nM PF543 for 12 h, followed by LPS treatment for 24 h. **a** Representative Western blots and quantitative data for iNOS, COX-2, TNF-a, IL-6, and GAPDH expression in each group of microglia. PF543 suppressed proinflammatory mediator release and prevented M1 microglia polarization in a concentration-dependent manner. **b** Immunofluorescence staining for COX-2 in each of group microglia (scale bar: 100 μm). **c** HAPI cells were treated with different concentrations of PF543 for 48 h and then the cell viability was evaluated by CCK-8 assay. *n* = 3 independent experiments. *P < 0.05 and **P < 0.01

To study the role of Sphk1 in neuroinflammation in vitro, we used PF543 to suppress Sphk1 activity. PF543 is a validated inhibitor of Sphk1 [22]. Western blot analysis of the expression of iNOS and COX-2, as well as the production of IL-6 and TNF-α in microglia, showed that PF543 pretreatment reduced expression levels of Sphk1 in a concentration-dependent manner in the treatment group compared with the control group (Fig. 4a, Control group vs. LPS group, *P* < 0.01). In the three groups for LPS and PF543 10 nM, PF543 100 nM groups, respectively, the iNOS rankings were 2.54 ± 0.11 > 2.01 ± 0.14 > 1.39 ± 0.06, LPS group vs. PF543 10 nM group, *P* = 0.028, PF543 10 nM group vs. PF543 100 nM group, *P* = 0.012; the COX-2 rankings were 3.14 ± 0.19 > 2.10 ± 0.19 > 1.36 ± 0.12, LPS group vs. PF543 10 nM group, *P* = 0.006, PF543 10 nM group vs. PF543 100 nM group, *P* = 0.036; the TNF-α rankings were 2.07 ± 0.05 > 1.67 ± 0.13 > 1.20 ± 0.10, PF543 10 nM group vs. PF543 100 nM group, *P* = 0.041, LPS group vs. PF543 100 nM group, *P* = 0.001; the IL-6 rankings were 1.93 ± 0.15 > 1.60 ± 0.12 > 1.13 ± 0.07, LPS group vs. PF543 100 nM group, *P* = 0.005).

These results were consistent with our immunofluorescence assay, which showed gradually declining staining for COX-2 in treated microglia (Fig. 4b, Control group vs. LPS group, *P* < 0.01. LPS group: 3.80 ± 0.29 vs. PF543 10 nM group: 2.69 ± 0.16, *P* = 0.003, PF543 10 nM group: 2.69 ± 0.16 vs. PF543 100 nM group: 2.01 ± 0.33, *P* = 0.048). To investigate potential PF543 cytotoxicity, we treated microglia with or without PF543 (concentrations: 0, 5, 10, 50, 100, and 500 nM and 1, 5, and 10 μM) for 48 h and evaluated the outcome of treatment by CCK-8 assay. We confirmed that PF543 did not cause significant cytotoxicity at concentrations below 1 μM (Fig. 4c, Control group: 1.11 ± 0.05 vs. 5 μM group: 0.86 ± 0.05, *P* = 0.017). These data indicate that blocking the activity of Sphk1 in microglia may attenuate M1 phenotype polarization and may safely be achieved with PF543.

### PF543 prevents neurons from undergoing M1 microglia-facilitated apoptosis

**Fig. 5:**
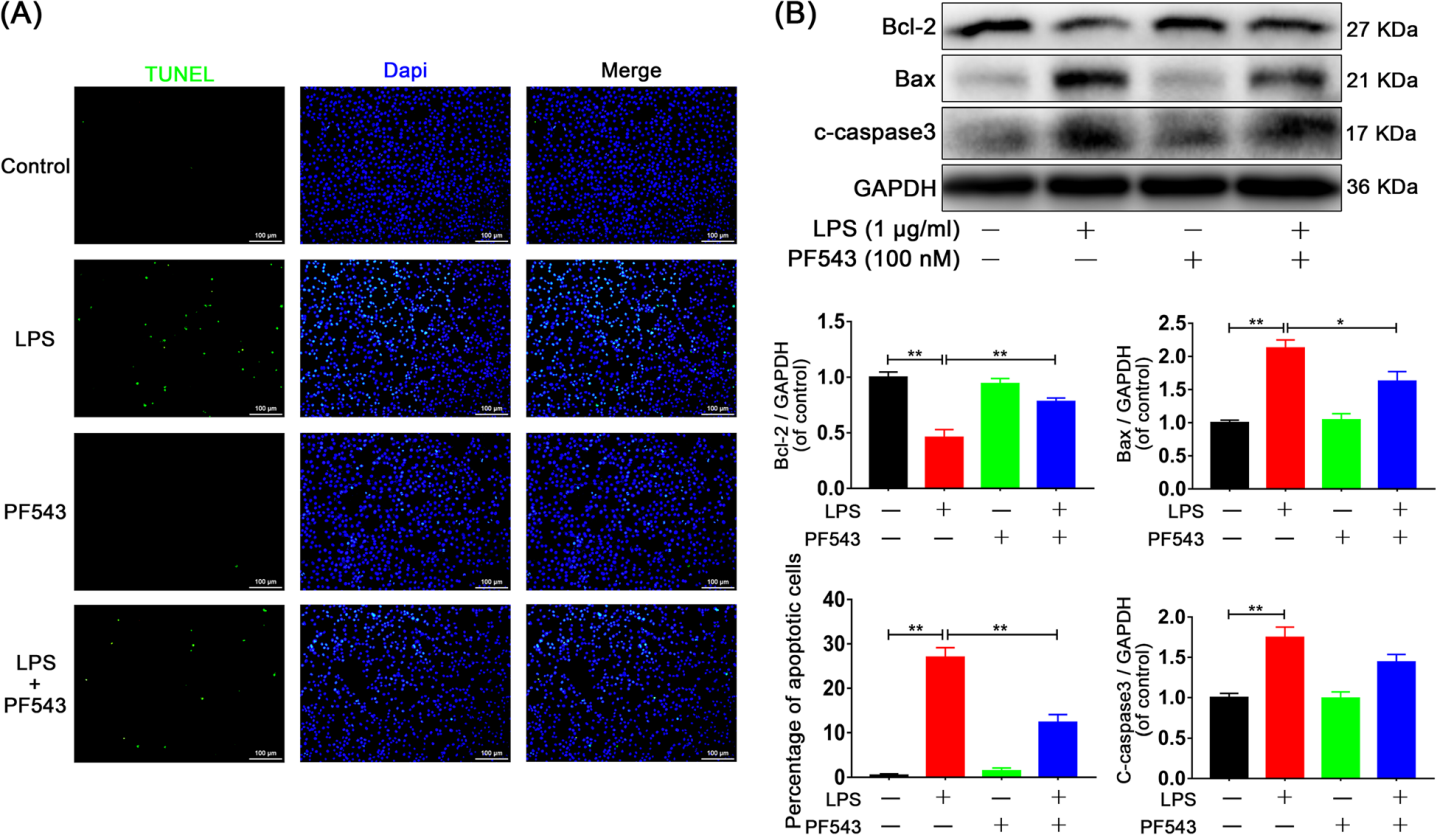
PF543 prevents M1 microglia-induced neuronal apoptosis. **a** TUNEL assay was performed in neurons cocultured with microglia (scale bar: 100 μm). The neuronal apoptosis induced by M1 microglia was significantly attenuated in cells cocultured with PF543 pretreated microglia. **b** Representative Western blots and quantitative data for Bcl-2, Bax, cleaved caspase 3, and GAPDH expression in each group of neurons. *n* = 3 independent experiments. *P < 0.05 and **P < 0.01

To further investigate whether neuronal apoptosis is related to Sphk1-mediated neuroinflammation, we established a microglia/neuron coculture system as previously mentioned with specific treatments. TUNEL assay was then performed to detect neuronal apoptosis. Results showed that PC12 cells cocultured with LPS-activated microglia underwent apoptosis at a significantly increased rate compared with cells in the control group. Moreover, cells that were cocultured with PF543-pretreated microglia underwent apoptosis at a significantly decreased rate compared with cells cocultured with M1 microglia with no pretreatment (Fig. 5a, Control group: 0.49 ± 0.29 vs. LPS group: 26.98 ± 2.16, *P* < 0.001, LPS group: 26.98 ± 2.16 vs. LPS + PF543 group: 12.32 ± 1.77, *P* < 0.001).

In addition, Western blot analysis showed the reduced expression of the apoptotic protein Bax and increased expression of the protective protein Bcl-2 in neurons cocultured with PF543-treated microglia. Expression of the apoptotic protein cleaved caspase-3 was non-significantly reduced in the PF543 pretreated coculture (Fig. 5b, Control group vs. LPS group, *P* < 0.01; LPS group vs. PF543 group: 0.46 ± 0.07 vs. 0.78 ± 0.03, *P* = 0.008 for Bcl-2, 2.13 ± 0.12 vs. 1.63 ± 0.15, P = 0.046 for Bax, 1.75 ± 0.13 vs. 1.44 ± 0.09, *P* > 0.05 for cleaved caspase 3). These results indicate that inhibition of Sphk1 in activated microglia can protect neurons against inflammation-facilitated apoptosis.

### Sphk1 mediates inflammation through NF-κB activation induced by S1P

**Fig. 6:**
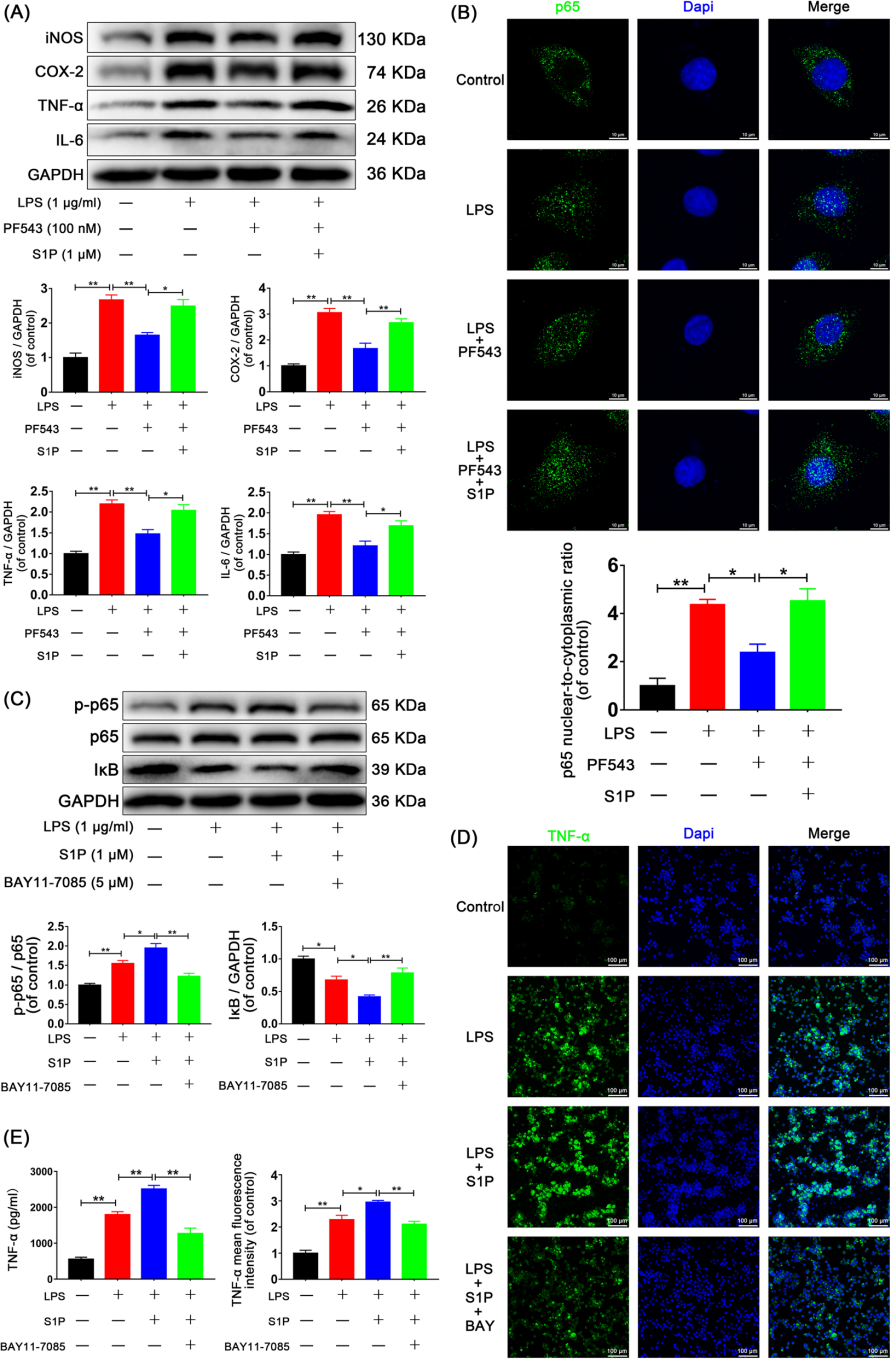
Sphk1-regulated neuroinflammation is related to S1P-induced NF-κB activation. HAPI cells were pretreated with PF543 for 12 h or BAY11-7085 for 2 h, followed by washing three times and treatment with LPS alone or LPS plus S1P for 24 h. **a** Representative Western blots and quantitative data for iNOS, COX-2, TNF-α, IL-6, and GAPDH expression. PF543 pretreatment attenuated LPS-induced M1 microglia polarization and proinflammatory cytokine production, effects that were reversed by addition of exogenous S1P. **b** Immunofluorescence staining for p65 was observed under confocal microscope (scale bar: 10 μm), and nuclear-to-cytoplasmic ratio of p65 was quantified. LPS-induced p65 nuclear translocation was suppressed by PF543 pretreatment and restored by addition of exogenous S1P. **c** Western blot analysis of p-p65, p65, IκB, and GAPDH expression in HAPI cells. Adding exogenous S1P amplified the NF-κB activation and inflammatory response, and BAY11-7085 significantly eliminated the amplification. **d** Immunofluorescence staining for TNF-α in each group of microglia (scale bar: 100 μm). **e** The secretion of TNF-α was also measured by ELISA assay. S1P promoted proinflammatory mediator release, and the effect was eliminated by BAY11-7085. *n* = 3 independent experiments. *P < 0.05 and **P < 0.01

Previous studies have demonstrated that NF-κB p65 activation is a required component of the inflammatory response and is, in part, regulated by intracellular and extracellular lipid molecules [31, 32]. To investigate the relationship between Sphk1 and inflammation, we examined the effects of PF543 and S1P on the NF-κB p65 activation. Western blot assays showed increased expression of iNOS, COX-2, TNF-α, and IL-6 after LPS treatment, which was inhibited by PF543 pretreatment and was then restored by exogenous S1P (Fig. 6a, Control group vs. LPS group, *P* < 0.01. LPS group vs. PF543 group: 2.67 ± 0.14 vs. 1.65 ± 0.07, *P* = 0.004 for iNOS, 3.06 ± 0.16 vs. 1.67 ± 0.21, *P* = 0.001 for COX-2, 2.20 ± 0.10 vs. 1.48 ± 0.10, *P* = 0.005 for TNF-α, 1.96 ± 0.08 vs. 1.21 ± 0.12, *P* = 0.003 for IL-6. PF543 group vs. S1P group: 1.65 ± 0.07 vs. 2.49 ± 0.19, *P* = 0.012 for iNOS, 1.67 ± 0.21 vs. 2.67 ± 0.15, *P* = 0.008 for COX-2, 1.48 ± 0.10 vs. 2.04 ± 0.14, *P* = 0.019 for TNF-α, 1.21 ± 0.12 vs. 1.69 ± 0.12, *P* = 0.032 for IL-6).

Through confocal observation of immunofluorescence staining, we found that NF-κB p65 significantly translocated from the cytoplasm to the nucleus after LPS treatment for 6 h and that this translocation was blocked by PF-543 pretreatment. Conversely, addition of exogenous S1P enhanced NF-κB p65 translocation (Fig. 6b, for the nuclear-to-cytoplasmic ratio, Control group vs. LPS group: 1.00 ± 0.31 vs 4.36 ± 0.23 *P <* 0.01. LPS group vs. PF543 group: 4.36 ± 0.23 vs 2.37 ± 0.36 *P* = 0.021. PF543 group vs. S1P group: 2.37 ± 0.36 vs 4.51 ± 0.52 *P* = 0.014). These data suggest that Sphk1-mediated neuroinflammation is dependent on the production of S1P, which then activates NF-κB p65 nuclear translocation.

To determine the relationship between S1P-induced NF-κB p65 activation and the inflammatory response, we used BAY11-7085, an inhibitor of NF-κB activation. Western blot assay for expression of phosphorylated-p65 (p-p65), p65, and IκB indicated that LPS enhanced p65 phosphorylation and IκB degradation. Adding exogenous S1P amplified NF-κB p65 activation, the effect of which was reversed by BAY11-7085 (Fig. 6c, Control group vs. LPS group: 1.00 ± 0.04 vs. 1.55 ± 0.07, *P* = 0.006 for p-p65/p65 ratio, 1.00 ± 0.04 vs. 0.68 ± 0.06, *P* = 0.012 for IκB; LPS group vs. S1P group: 1.55 ± 0.07 vs. 1.95 ± 0.12, *P* = 0.034 for p-p65/p65 ratio, 0.68 ± 0.06 vs. 0.42 ± 0.03, *P* = 0.040 for IκB; S1P group vs. BAY group: 1.95 ± 0.12 vs. 1.22 ± 0.08, *P* = 0.001 for p-p65/p65 ratio, 0.42 ± 0.03 vs. 0.78 ± 0.08, *P* = 0.006 for IκB).

Using immunofluorescence analysis for TNF-α, we found that S1P facilitated the release of this proinflammatory mediator, and BAY11-7085 countered this effect (Fig. 6d, Control group vs. LPS group, *P* < 0.01. LPS group: 2.28 ± 0.16 vs. S1P group: 2.95 ± 0.06, *P* = 0.015, S1P group: 2.95 ± 0.06 vs. BAY group: 2.11 ± 0.11, *P* = 0.004, BAY group: 2.11 ± 0.11 < LPS group: 2.28 ± 0.16). ELISA assay also indicated that the secretion of TNF-α was significantly induced 24 h after LPS stimulation, and S1P treatment increased LPS-induced secretion of TNF-α, while BAY11-7085 antagonized its effects (Fig. 6e, Control group vs. LPS group, *P* < 0.001. LPS group: 1791.99 ± 87.23 vs. S1P group: 2505.69 ± 104.48, *P* = 0.007. S1P group: 2505.69 ± 104.48 vs. BAY group: 1266.61 ± 154.47, *P* < 0.001). These findings indicate that S1P-induced NF-κB p65 activation, which may result from extracellular regulation, may play a crucial role in the inflammatory amplification cascade.

### Inhibition of Sphk1 decreases the expression of S1PR3 and phosphorylation of p38 MAPK analysis in vivo and in vitro

**Fig. 7:**
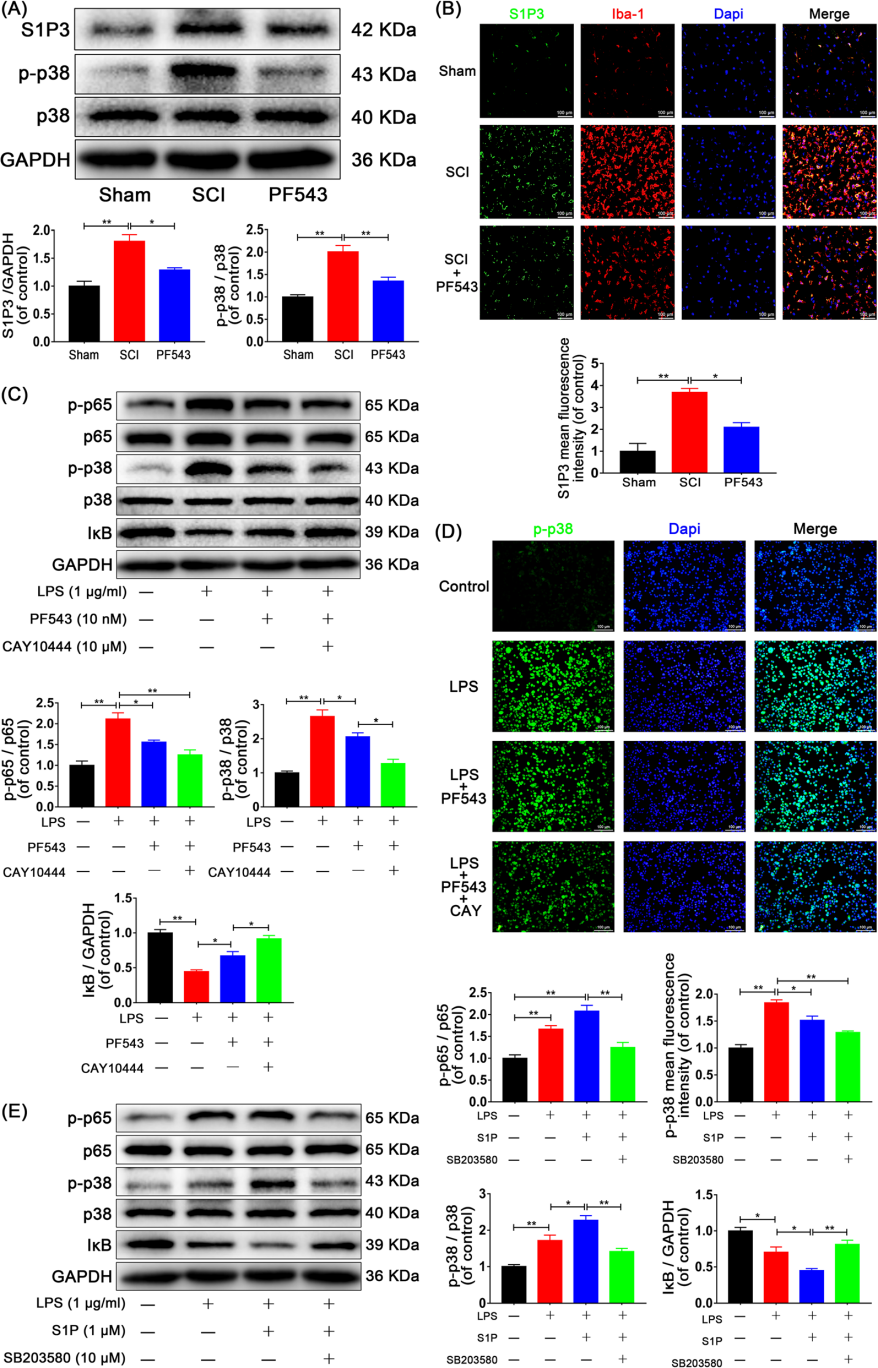
S1P/S1PR3/p38 pathway contributes to Sphk1-related p65 activation. For in vivo experiments, rats were sacrificed and specimens were taken at 3 days after SCI. *n* = 3 per group. **a** Representative Western blots and quantitative data for S1PR3, p-p38, p38, and GAPDH expression in each group of rats. S1PR3 expression and p38 phosphorylation were enhanced in the SCI group and were inhibited by PF543 treatment. **b** Double-staining for S1PR3 (green)/Iba-1 (red) in injured spinal cord tissue from each group of rats (scale bar: 100 μm). For in vitro studies, HAPI cells were pretreated with PF543 for 12 h and CAY10444 for 2 h or SB203580 for 2 h, followed by washing three times and treatment with LPS alone or LPS plus S1P for 24 h, *n* = 3 independent experiments. **c** Representative Western blots and quantitative data for p-p65, p65, p-p38, p38, IκB, and GAPDH expression in each group of microglia. Both Sphk1 and S1PR3 activity influenced the activation of p65 and p38. **d** Immunofluorescence staining for p-p38 in each of group microglia (scale bar: 100 μm), PF543 pretreatment decreased p38 phosphorylation, and inhibition of S1PR3 strengthened the inhibitory effect. **e** Western blot analysis of p-p65, p65, p-p38, p38, IκB, and GAPDH expression in each group of microglia. S1P enhanced the activation of p65 and p38 induced by LPS stimulation, the effect was reversed by SB203580. *P < 0.05 and **P < 0.01

To elucidate the signaling pathways associated with Sphk1 in more detail, we hypothesized that both the activation of Sphk1 and subsequent S1P binding on surface receptors are required for the effects of downstream inflammatory molecules. In our in vivo study, Western blot analysis demonstrated that levels of S1PR3 expression and p38 MAPK phosphorylation were significantly increased in the SCI group compared with that of the sham group and were decreased in the PF543-treated group compared with that of the SCI group (Fig. 7a, Sham group vs. SCI group: 1.00 ± 0.09 vs. 1.80 ± 0.12, *P* = 0.002 for S1PR3, 1.00 ± 0.05 vs. 2.01 ± 0.14, *P* < 0.001 for p-p38/p38 ratio; SCI group vs. PF543 group: 1.80 ± 0.12 vs. 1.29 ± 0.04, *P* = 0.015 for S1PR3, 2.01 ± 0.14 vs. 1.35 ± 0.09, *P* = 0.008 for p-p38/p38 ratio). Double-immunofluorescence staining for S1PR3 and Iba-1 showed increased S1PR3 expression in the SCI group compared with the sham group, and staining intensity decreased in the PF543-treatment group (Fig. 7b, Sham group: 1.00 ± 0.36 vs. SCI group: 3.69 ± 0.18, *P* < 0.001; SCI group: 3.69 ± 0.18 vs. PF543 group: 2.10 ± 0.21, *P* = 0.012).

Next, our group explored whether NF-κB activation is associated with the S1P/S1PR3/p38 MAPK pathway in vitro. Microglia were pretreated with CAY10444, a selective antagonist of S1P binding to S1PR3, in addition to PF543 pretreatment and LPS treatment. Expression levels of p38, phospho-p38, p65, phospho-p65, and IκB were analyzed by Western blot as shown in Fig. 7c (Control group vs. LPS group, *P* < 0.01 for p-p65/p65 ratio, p-p38/p38 ratio, and IκB. LPS group vs. PF543 group: 2.11 ± 0.15 vs. 1.56 ± 0.05, *P* = 0.032 for p-p65/p65 ratio, 2.65 ± 0.19 vs. 2.06 ± 0.12, *P* = 0.049 for p-p38/p38 ratio, 0.44 ± 0.03 vs. 0.67 ± 0.06, *P* = 0.039 for IκB; PF543 group vs. CAY group: 2.06 ± 0.12 vs. 1.27 ± 0.12, *P* = 0.012 for p-p38/p38 ratio, 0.67 ± 0.06 vs. 0.92 ± 0.05, *P* = 0.026 for IκB; LPS group vs. CAY group: 2.11 ± 0.15 vs. 1.25 ± 0.12, *P* = 0.003 for p-p65/p65 ratio). The results indicated that the activation of p38 MAPK and NF-κB p65 was enhanced after LPS stimulation, PF543 pretreatment reduced phosphorylation of p38 MAPK and NF-κB p65 and reduced degradation of IκB when compared to no pretreatment. Moreover, the inhibition of p38 MAPK and NF-κB p65 were strengthened in the group treated with CAY10444 2 h prior to LPS. We also performed immunofluorescence analysis for p-p38 in microglia, and consistent results were observed (Fig. 7d, Control group: 1.00 ± 0.06 vs. LPS group: 1.84 ± 0.06, *P* < 0.001; LPS group: 1.84 ± 0.06 vs. PF543 group: 1.51 ± 0.08, *P* = 0.020; LPS group: 1.84 ± 0.06 vs. CAY group: 1.29 ± 0.03, *P* < 0.001).

We next detected NF-κB p65 activity via another inhibitor, SB203580, which selectively acts on p38 MAPK. Western blot analysis showed that activation of p38 MAPK and NF-κB p65 induced by LPS was amplified by S1P, and the amplification effect was inhibited by SB203580 (Fig. 7e, Control group vs. LPS group, *P* < 0.05; LPS group vs. S1P group: 1.67 ± 0.08 vs. 2.08 ± 0.13, *P* > 0.05 for p-p65/p65 ratio, 1.72 ± 0.15 vs. 2.27 ± 0.13, *P* = 0.030 for p-p38/p38 ratio, 0.70 ± 0.07 vs. 0.45 ± 0.03, *P* = 0.042 for IκB; S1P group vs. SB group: 2.08 ± 0.13 vs. 1.25 ± 0.11, *P* = 0.002 for p-p65/p65 ratio, 2.27 ± 0.13 vs. 1.42 ± 0.08, *P* = 0.003 for p-p38/p38 ratio, 0.45 ± 0.03 vs. 0.81 ± 0.06, *P* = 0.006 for IκB). Collectively, these findings indicate that S1P acting on S1PR3 enables NF-κB p65 transcription through the p38 MAPK pathway, which participates in Sphk1-mediated neuroinflammation in SCI.

## Discussion

Our investigation discovered the role of Sphk1 in traumatic SCI neuroinflammation and demonstrated that the Sphk1/S1P axis is a feasible target for mediating neuronal injury and improving neurological outcomes after SCI. The role of the Sphk1/S1P/S1PR pathway in CNS injury and neuroinflammation is complex, and most of the current literature base has focused on neurodegenerative diseases such as multiple sclerosis, Alzheimer’s disease, and Parkinson’s disease. The role of this pathway in traumatic SCI remained rarely described. Our study uniquely uncovers the balance between sphingolipid metabolism and the inflammatory response in traumatic SCI. These findings offer a preclinical proof of concept that signaling by a sphingolipid may be an effective target to control the inflammation of traumatic SCI and improve functional outcome. This study primarily addresses the mechanism of neuroinflammation by which secondary injury to neurons progresses after SCI, with a focus on the effect of pharmacological inhibition of Sphk1 on microglial phenotype differentiation and proinflammatory factor synthesis.

**Fig. 8:**
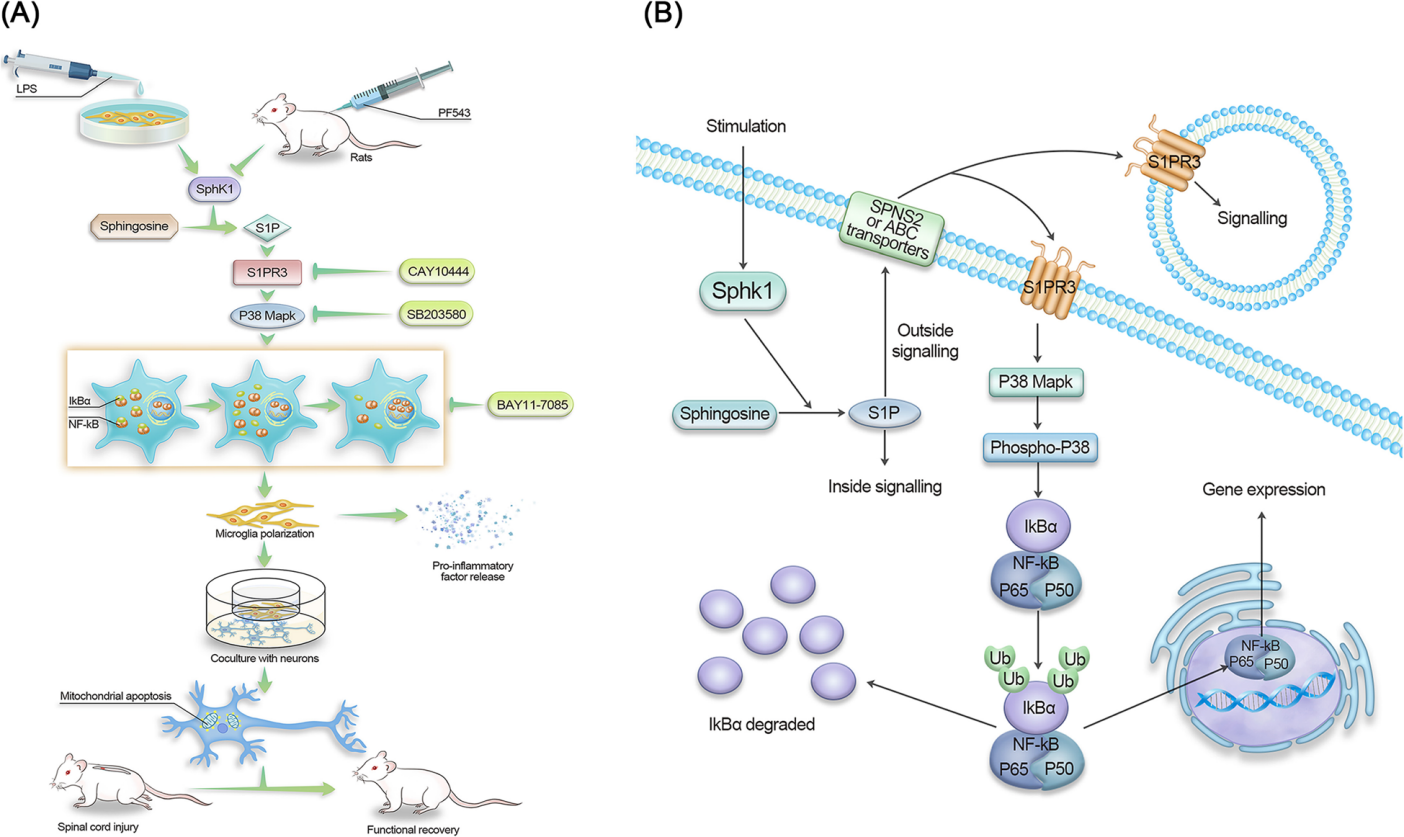
**a** Schematic of the S1P/S1PR3/p38 MAPK signaling pathway involved in neuroinflammation. After LPS stimulation or spinal cord injury, resting microglia underwent M1 polarization and released proinflammatory mediators to induce neuronal apoptosis, which was improved by the specific inhibitors. **b** One of the Sphk1/S1P signaling pathways. S1P catalyzed by Sphk1 in the cytoplasm are transported outside the cell and bind to membrane receptors (S1PR3), then downstream activation of p38 MAPK phosphorylation and NF-κB nuclear transcription is triggered

Amplification of the inflammatory response is an important cause of excessive secondary neuronal injury and poor functional outcomes in SCI. Just as autophagy is one of the important means of regulating intracellular homeostasis, lipid metabolism alters the homeostasis of inflammation [31, 32]. Our study demonstrated that the onset of inflammation was associated with abnormal lipid metabolism and that alterations in lipid metabolism at the molecular level could attenuate the inflammatory response. Specifically, we demonstrated a tendency of microglial M1 polarization and an increase in Sphk1 expression after SCI, which corresponded with incremental proinflammatory cytokine production. Subsequently, inhibition of Sphk1 attenuated microglial M1 polarization and proinflammatory cytokine production in vitro and in vivo after SCI. PF543, the intense Sphk1 inhibitor, which can block the activity of Sphk1 and reduce the intracellular level of S1P and its secretion [30], significantly reduced inflammatory markers post-SCI and exhibited anti-inflammatory properties in a concentration-dependent manner in vitro (Fig. 8a). These findings reinforce that the activity of Sphk1 and the production of S1P are closely related to the specific state of inflammation in acute spinal cord injury, uncovering a new network between lipid metabolism and the neuroinflammatory response in this particular pathology. We further demonstrated that this pathway can be targeted and attenuated, resulting in regulation of the inflammatory response and improved functional recovery following acute SCI. Future study should investigate longer-term recovery for further clinical translation.

Many studies have shown that PF543 affects Sphk1 activity, yet data about the pharmacokinetics of PF543, such as absorption efficiency, drug concentration in the blood, hepatic and renal metabolic rates, and drug toxicity, have not been described in detail [22, 30, 33, 34]. Although pharmacokinetics of PF543 still need to be clarified in in vivo experiments, one study concluded that a dose of PF543 at 10 mg/kg every 2 days in animal experiments is sufficient for pharmacological inhibition of Sphk1 [22]. Therefore, our animal studies adopted the drug administration dose at 10 mg/kg/d and also demonstrated efficacy. Additionally, prior study of the pharmacokinetics of PF543 in mice demonstrated a half-life of 1.2 h in blood samples of mice dosed intraperitoneally with 10 mg/kg of PF543 for 24 h and that this dosing resulted in a blood concentration of 50 μg/ml [35]. Important future study should pursue more intense investigation of the pharmacokinetics and metabolism of PF543 for further translation.

Sphk1 has been implicated as an essential intracellular messenger regulating the neuroinflammatory response that has been studied and reported at length, but the detailed functions of Sphk1 remain a matter of debate and likely differ in varying disease pathologies. Activation of Sphk1 leads to subsequent production of S1P, which has been implicated in transcription factor activation and inflammatory response in diseases such as asthma, inflammatory bowel disease, and sepsis [36–38]. It has been proposed that LPS plus S1P treatment intensifies leukocyte adhesion and proinflammatory responses in endothelial cells [39]. Microglial activation and Sphk1 induction have been identified as a major cellular source of acute ischemic injury and neuroinflammation in stroke [40]. Neural cells, including microglia, were found to express S1P receptors (S1PRs), making them a target of S1P in the CNS [18, 41]. Ultimately, understanding of this inflammatory cascade in the pathology specific to SCI has remained the limiting factor in identifying a useful biological target for treatment, and our study has taken the first steps at answering this critical question.

In addition to inflammation-mediated secondary injury, M1 microglia can also trigger neuronal mitochondrial dysfunction and promote neuronal apoptosis [27]. SCI resulted in increased expression of apoptosis-related proteins cleaved caspasae-3 and Bax, as well as reduced expression of the anti-apoptotic protein Bcl-2. This study shows that Sphk1 suppression in microglia attenuated neuronal apoptosis and resulted in improved motor function recovery at 2 weeks post-injury. It is important to note that our study places focus on the role of microglia in the S1P neuroinflammatory pathway post-SCI. Other functions of microglia, such as phagocytosis, neural restoration, and the functions of astrocytes, oligodendrocytes, neurons, and vascular endothelial cells, which are closely related to SCI and repair, are also regulated by Sphk1/S1P. Therefore, this study can be considered as a beginning to the exploration of the involvement of Sphk1/S1P in the regulatory mechanisms of CNS trauma. Future research will need to investigate the expression pattern of Sphk1 and the impact of this pathway on other cell types after traumatic SCI.

At the level of transcriptional regulation, studies have confirmed that the inflammatory response, including inflammatory cytokine production, is orchestrated by transcription factors, such as NF-κB [42, 43] and Stat3 [36]. Conflicting studies have demonstrated that S1P induces NF-κB activation [14, 44] and that HDL-bound S1P attenuates cytokine-induced NF-κB [45], suggesting that the effects of S1P are variable and dependent on cofactors and substrate binding. In the present in vitro study, we demonstrated that phosphorylation of NF-κB p65 and degradation of IκB is present in M1 microglia, that S1P increases nuclear translocation of NF-κB p65, and that this translocation can be attenuated by pharmacological inhibition of Sphk1, leading to lower expression of inflammatory phenotype proteins and proinflammatory mediators. Suppression of NF-κB translocation mitigated the amplification effect of NF-κB activation and the proinflammatory cytokine production induced by S1P (Fig. 8a). These findings indicate that the production of S1P via the Sphk1 neuroinflammatory pathway in M1 microglia enhances NF-κB nuclear translocation resulting in a downstream neuroinflammatory response, and this nuclear translocation can be attenuated by inhibiting the production of S1P via inhibition of Sphk1.

It has been previously demonstrated that S1P is generated intracellularly by sphingosine kinase at the plasma membrane in response to multiple growth factors and cytokines, acting on as-yet-unidentified intracellular targets. An active transport mechanism also allows for S1P to act extracellularly, via S1P receptors (S1PRs) [46]. S1P/S1PR signaling plays a crucial role in the regulation of many pathophysiological processes, such as acute liver failure [47], tumor cell migration [48], Alzheimer’s disease [49], and proliferation of pericytes in SCI [17]. However, the mechanism by which Sphk1/S1P/S1PRs regulate neuroinflammation in traumatic SCI is unclear. Several recent studies have focused on S1PR3 as a critical receptor in regulating inflammation [50, 51], contributing to our choice to focus on this receptor in the present study. Studies have shown that S1PR3 is involved in RhoA activation and induces transcription of the inflammatory mediators COX-2, IL6, and VEGFa [50]. Given the deleterious effects of inflammation post-SCI and the described links of this subtype with inflammatory markers, the S1PR3 subtype was chosen as the focus of the present study. Moreover, a study further ensured that S1PR3 activation is closely associated with the M1 polarization of activated microglia in the ischemic brain [19]. While future study may investigate the potential crosstalk and associated roles of other S1P receptors, the present study adds to this body of literature in confirming the role of S1PR3 in spinal cord-mediated inflammation, supporting our hypothesis that the Sphk1/S1P/S1PR3 axis is responsible for neuroinflammatory response.

The present in vivo study discovered the neuroinflammatory signaling pathway of Sphk1/S1P/S1PR3 in SCI (Fig. 8b). We demonstrated that S1PR3 expression was clearly upregulated in activated M1 microglia in vivo. We also discovered that phosphorylation of p38 MAPK was increased in M1 microglia of SCI rats, suggesting that p38 MAPK may be a downstream effector influenced by S1P/S1PR3 binding. This p38 phosphorylation was reduced by inhibiting Sphk1. By using the selective S1PR3 antagonist CAY10444 (Fig. 8a) combined with the Sphk1 inhibitor PF543, we confirmed a further reduction in p38 MAPK phosphorylation and NF-κB p65 phosphorylation, suggesting that phosphorylation/activation of these factors is influenced by Sphk1 activity and subsequent interaction of S1P at S1PR3. To further investigate the crosstalk between p38 MAPK phosphorylation and S1P-induced NF-κB activation in this pathway (Fig. 8a), we demonstrated that exogenous S1P amplified the phosphorylation of p38 MAPK and NF-κB p65 and that this effect could be inhibited by a specific p38 MAPK inhibitor. Future study will be needed to further understand the contribution of intracellular S1P activity on NfkB activation; however, the present study has exposed the significant role of exogenous S1P in the described inflammatory cascade after traumatic SCI, suggesting that Sphk1 activation and subsequent S1P binding to S1PR3 are important steps in the initial activation of the neuroinflammatory pathway, which then leads to phosphorylation of p38 MAPK and promotes NF-κB p65 nuclear translocation leading to amplification of the inflammatory cascade in SCI.

These findings unravel the important role of the S1P/S1PR3/p38 MAPK pathway in the secondary neuroinflammatory response of acute spinal cord injury and the role of dysregulated lipid metabolism in this pathology. Contributing a novel understanding of the molecular mechanisms of the inflammatory cascade in this specific pathology now provides a new translational implication for targeted drug therapies in SCI. We have demonstrated the feasibility of manipulating this pathway via specific Sphk1 inhibitors to successfully alter the inflammatory cascade and improve functional outcomes. Future investigation should focus on the ideal limb of this cascade for inhibition and intervention and translational routes of administration. Therapies that target Sphk1/S1P/S1PR are complex and multifaceted and may in the future replace the current inefficient conservative therapies such as NSAIDs and corticosteroids while avoiding the invasiveness of surgical intervention, with the hope of providing the first targeted biologic therapy for the treatment of acute SCI.

Sphingomyelin is abundant in the nervous system, and S1P receptors are widely expressed in various cells, with many cell responses depending on the Sphk1/S1P/S1PRs axis. For instance, one study demonstrated that localized delivery of FTY720 can reduce pathological astrogliosis by targeting the S1P1 receptor of astrocytes and provide a new therapeutic strategy for SCI treatment [52]. Thus, our future study will further investigate the expression of S1P receptors in cells other than microglia, such as astrocytes and neurons, and verify their possible synergistic effects in the neuroinflammatory process. In addition to the SphK1/S1P/S1PRs axis, Sphk1 may have other regulatory pathways. When neuronal autophagy is pharmacologically stimulated, Sphk1 re-localizes to the endocytic and autophagic organelles, suggesting that autophagy in neurons is also regulated by Sphk1 [53]. Sphingosine kinase 1 may be involved in other mechanism pathways, such as sphingosine kinase 1 cooperates with autophagy to maintain endocytic membrane trafficking [54]. Thus far, explorations targeting Sphk1 and its links to autophagy, inflammation, and central neuropathy are rarely reported, providing a possible avenue for breakthroughs in our future study.

## Conclusions

In conclusion, our study has uncovered the role of abnormal lipid metabolism via the Sphk1 cascade as an etiology of secondary inflammatory injury after traumatic SCI. SCI enhances Sphk1 expression and activity in injured spinal cord tissue. Sphk1 modulates microglial M1 polarization and proinflammatory cytokine production via intracellular S1P signaling, as well as extracellular S1P binding to S1PR3, with ultimate phosphorylation of p38 MAPK and nuclear translocation and activation of NF-κB p65. This S1PR3/p38 MAPK pathway results not only in the release of proinflammatory molecules, but also results in loss of neuronal cells via apoptosis. Inhibition along the Sphk1/S1P/S1PR3 axis is feasible, reduces neuronal apoptosis, improves neurological functional recovery, and is presented as a translational therapeutic target for mitigating the inflammatory cascade amplification that exacerbates secondary injury in SCI.

## Data Availability

All data supporting the conclusions of this manuscript are provided in the text and figures. Please contact the author for data requests.

## Abbreviations

Sphk1: Sphingosine kinase 1
S1P: Sphingosine-1-phosphate
S1PR: Sphingosine-1-phosphate receptor
S1PR3: Sphingosine-1-phosphate receptor subtype 3
SCI: Spinal cord injury
LPS: Lipopolysaccharide
DMEM: Dulbecco’s modified Eagle’s medium
FBS: Fetal bovine serum
CCK-8: Cell Counting Kit-8
PBS: Phosphate-buffered solution
DMSO: Dimethyl sulfoxide
DAPI: 4',6-Diamidino-2-phenylindole
TBST: Tris-buffered saline with Tween
WB: Western Blot
TUNEL: Terminal-deoxynucleoitidyl Transferase Mediated Nick End Labeling
HE: Hematoxylin-eosin staining
ANOVA: Analysis of variance
BBB: Basso, Beatlie, Bresnahan
iNOS: Inducible nitric oxide synthase
COX-2: Cyclooxygenase-2
IL-6: Interleukin-6
TNF-α: Tumor necrosis factor-α
Bcl-2: B cell lymphoma-2
Bax: Bcl-2 Associated X Protein
CC-3: Cleaved caspase-3
NF-κB: Nuclear factor κB
IκB: NF-κB inhibitor
MAPK: Mitogen-activated protein kinase
GAPDH: Glyceraldehyde-3-phosphate dehydrogenase
Iba-1: Ionized calcium-binding adapter molecule 1
Neun: Neuronal nuclei
NGF: Nerve growth factor
